# Normal-Weight Obesity and an Unfavorable Cardiometabolic Profile: Results from the Study of Workers’ Health (ESAT)

**DOI:** 10.3390/healthcare14081008

**Published:** 2026-04-11

**Authors:** Fernando Gomes de Jesus, Alice Pereira Duque, Grazielle Vilas Bôas Huguenin, Mauro Felippe Felix Mediano, Maicon Teixeira de Almeida, Carla Christina Ade Caldas, Silvio Rodrigues Marques-Neto, Luiz Fernando Rodrigues Junior

**Affiliations:** 1 Department of Physiological Sciences, Biomedical Institute, Federal University of the State of Rio de Janeiro, Rio de Janeiro 22290-240, Brazil; fernando.jesus@edu.unirio.br (F.G.d.J.); alice-duque@hotmail.com (A.P.D.); luiz.junior@unirio.br (L.F.R.J.); 2 Education and Research Department, National Institute of Cardiology, Rio de Janeiro 22240-006, Brazil; ghuguenin@id.uff.br (G.V.B.H.); mffmediano@gmail.com (M.F.F.M.); 3 Nutrition and Dietetics Department, Fluminense Federal University, Niterói 24020-140, Brazil; 4 Evandro Chagas National Institute of Infectious Diseases, Oswaldo Cruz Foundation, Rio de Janeiro 21040-900, Brazil; 5 Programa de Pós-Graduação em Ciências da Atividade Física (PPGCAF/UNIVERSO), Universidade Salgado de Oliveira, Niterói 24030-060, Brazil; maicon_teixeira_almeida@hotmail.com (M.T.d.A.); carlaade41@gmail.com (C.C.A.C.); 6 Programa de Pós-Graduação em Biologia Humana e Experimental (BHEx/UERJ), Universidade do Estado do Rio de Janeiro, Rio de Janeiro 20551-030, Brazil; 7 Programa de Pós-Graduação em Fisiopatologia Clínica e Experimental (FISCLINEX), Laboratório de Pesquisas Clínicas e Experimentais em Biologia Vascular (BioVasc), Rio de Janeiro 20551-030, Brazil

**Keywords:** normal weight obesity, autonomic function, heart rate variability, cardiometabolic risk, cardiovascular risk

## Abstract

**Highlights:**

**What were the main findings of this study?**

- Individuals with normal body mass index (BMI) but elevated body fat (NWO) exhibited worse lipid profiles, hemodynamic parameters, autonomic imbalance, and higher cardiometabolic risk scores than NWNB.
- The NWO phenotype was associated with selected cardiometabolic markers after adjustment, including low-density lipoprotein cholesterol (LDL-c), triglycerides, and autonomic indices. Body composition findings should be interpreted as confirmatory of the phenotype rather than as independent markers.

**What are the implications of the main findings?**

- Body mass index (BMI) alone may underestimate cardiometabolic risk, reinforcing the importance of incorporating body composition assessment in clinical and epidemiological settings. The identification of NWO individuals may help refine cardiometabolic risk screening in apparently normal-weight populations, although prospective studies are needed to determine its predictive value for future events.

**Abstract:**

Background: Normal-weight obesity (NWO) is a nutritional status in which individuals have a normal body mass index (BMI) with a high percentage of body fat (%BF). However, the impact of elevated %BF on cardiometabolic risk remains unclear. This study aimed to evaluate whether NWO is associated with worse cardiometabolic risk markers and scores. Methods: We conducted a cross-sectional study using a convenience sample of employees from a public hospital. Participants aged ≥18 years with a BMI between 18.5–24.9 kg/m^2^ were included in the study. %BF was categorized according to sex and age (InBody720). Normal weight and normal %BF (NWNB) and NWO were defined using cutoff points. Body composition, serum biochemical and inflammatory markers, hemodynamics, and autonomic function were considered cardiometabolic risk markers. The visceral fat area (VFA), atherogenic coefficient (AC), atherogenic index of plasma (AIP), body shape index (ABSI), and Framingham Risk (FR) score were considered cardiometabolic risk scores. Statistical significance was set at *p* < 0.05. Results: Of the 228 eligible participants, 52 met the inclusion criteria (NWNB, N = 29 and NWO, N = 23). Participants with NWO presented worse values of lipid profiles, anthropometric measurements, hemodynamic parameters, and autonomic function indices. After adjustment for age and sex, NWO remained associated with selected cardiometabolic markers, particularly LDL-c, triglycerides, and autonomic indices, whereas body composition findings should be interpreted as confirmatory of the phenotype. Conclusions: In this cross-sectional secondary analysis, NWO was associated with worse cardiometabolic markers and selected risk scores compared with NWNB. These findings support an unfavorable cardiometabolic profile in individuals with NWO, but do not allow inferences about future cardiometabolic events or causal relationships. Longitudinal studies are needed to clarify its prognostic significance.

## 1. Introduction

Obesity is a chronic, multifactorial, and complex disease associated with increased global morbidity and mortality rates [1]. Projections suggest that by the year 2030, approximately one billion individuals worldwide will be obese, with developed and developing nations bearing the brunt of this escalating challenge [2].

The increase in body fat percentage that occurs in obesity reflects the accumulation of adipose tissue, predominantly in the visceral region, leading to a chronic pro-inflammatory state characterized by the elevated release of pro-inflammatory cytokines, such as tumor necrosis factor-alpha (TNF-α) and interleukin-6 (IL-6), which amplifies insulin resistance and accelerates atherogenic processes, thus increasing the risk of cardiovascular events [3,4]. Overweight and obesity are also associated with autonomic nervous system (ANS) dysfunction, characterized by heightened sympathetic activity and/or diminished parasympathetic regulation (also associated with cardiovascular risk) [5].

The World Health Organization (WHO) defines obesity as the abnormal or excessive accumulation of adipose tissue, which increases health risks. Although the diagnosis of obesity relies on body mass index (BMI; ≥30 kg/m^2^), this definition often fails to identify individuals with abnormal body composition [6]. In this context, normal-weight obesity (NWO) describes individuals with eutrophic BMI (between 18.5 and 24.9 kg/m^2^) but an elevated percentage of body fat, which has been associated with cardiometabolic alterations [7,8,9].

The NWO has been previously linked to cardiometabolic dysregulation, hypertension, type 2 diabetes (DM2), dyslipidemia, increased inflammatory markers, and cardiac dysfunction [7,8,9]. Nevertheless, studies directly assessing cardiometabolic risk markers and scores in individuals with NWO compared to those with normal weight and normal body fat (NWNB) remain scarce. Therefore, this study aimed to evaluate whether the NWO phenotype is associated with worse cardiometabolic risk markers and scores, and should be interpreted as an exploratory, hypothesis-generating analysis conducted in a specific occupational cohort of hospital workers, rather than as evidence applicable to the general population.

## 2. Materials and Methods

### 2.1. Study Design

This cross-sectional study is a secondary analysis of the comprehensive database of an observational study entitled “Evaluation of stress indicators, body composition, and metabolic profile in employees of a cardiology reference hospital: contributions to quality of life—the Worker Health Study (ESAT),” conducted at the National Institute of Cardiology, Rio de Janeiro, Brazil (NIC), from November 2018 to March 2020. The inclusion criteria of the ESAT study were as follows: being an active employee of NIC and being aged ≥18 years. The exclusion criteria were as follows: being on medical sick leave, being assigned to another healthcare unit, having undergone recent surgery, fasting for more than 13 h, being pregnant and/or lactating, not responding to the team’s attempts to contact for data collection (minimum of three attempts), or undergoing the second day of collection (D2) after more than two months from the first day of collection (D1).

We used a convenience sample from this ESAT database, without calculating a specific sample size, making use of data that had already been collected prospectively [10]. Participants with a body mass index (BMI) between 18.5 and 24.9 kg/m^2^ were included in the study. Participants with missing information for the characterization of the anthropometric profiles were excluded from the study. The present study followed the recommendations of STROBE (Strengthening the Reporting of Observational Studies in Epidemiology), approved by ethics committee of the National Institute of Cardiology (CAAE: 96222718.7.0000.5272/Opinion: 5.046.117).

### 2.2. Anthropometric and Body Composition Assessment

Anthropometric data included body weight, height, body mass index (BMI), waist, hip, and neck circumferences, in accordance with World Health Organization (WHO) recommendations, as described by Araújo et al. [1,10]. BMI was calculated as weight (kg) divided by height squared (m^2^) [10]. The InBody 720^®^ octapolar segmental direct multifrequency bioimpedance analyzer (Biospace, Seoul, Republic of Korea) was used to measure weight in kilograms (kg), body fat percentage (%BF), and visceral fat area (VFA). Circumference measurements were performed using a non-elastic tape with millimeter precision. Waist circumference (WC) was measured during expiration between the midpoint of the last rib and the iliac crest. Hip circumference (HC) was measured at the point of greatest apparent HC. Neck circumference (NC) was measured at the level of the cricothyroid cartilage.

Volunteers were categorized by %BF based on sex and age, as shown in Table S1. Individuals with %BF values appropriate for their age and sex were considered NWNB. Those with %BF values above the expected range were classified as NWO.

### 2.3. Biochemical and Inflammatory Markers Assessment

Serum biochemical analysis was performed using blood samples collected in the morning after a 12-h fast. Serum concentrations of glucose, triglycerides (TG), total cholesterol (TC), low-density lipoprotein cholesterol (LDL-c), high-density lipoprotein cholesterol (HDL-c), and C-reactive protein (CRP) were determined. Glycemia was measured using the hexokinase method; TG, TC, and HDL-c were assessed by enzymatic colorimetric methods; LDL-c by immunoassay; and CRP by immunoturbidimetric assay.

### 2.4. Hemodynamics Assessment

Hemodynamic variables were assessed through systolic and diastolic blood pressure (SBP and DBP, respectively) measurements, which are expressed in mmHg. The mean arterial pressure (MAP) was estimated as a weighted average calculated from the SBP with a weight of 1 and the DBP with a weight of 2. The double product (obtained by multiplying the SBP by the heart rate) was measured. Blood pressure was measured on the right upper limb of all participants in the supine position using a digital sphygmomanometer (MA100, G-Tech^®^, Onbo Electronic, Shenzhen, China).

### 2.5. Autonomic Function Assessment

Autonomic assessment was conducted with the volunteers in the supine position with the head elevated at a 30° angle for 15 min. Subsequently, they were instructed to assume the orthostatic position for 3 min. Throughout the entire protocol duration (18 min), the volunteers were monitored using a portable electrocardiograph ECGV6^®^ (HW Sistemas, HeartWare Ltd., Framingham, MA, USA) and a Polar^®^ heart rate monitor (V800). Blood pressure was measured at the beginning, after 15 min, and at the end of the test using a digital sphygmomanometer (G-Tech^®^). This protocol enabled the assessment of heart rate variability (HRV), the orthostatic test, and the orthostatic hypotension test (OHT).

RR intervals obtained by the Polar V800^®^ (Polar Electro Oy, Kempele, Finland) heart rate monitor were utilized for heart rate variability (HRV) analysis using the Kubios^®^ software (v. 2.2, UEF, Kuopio, Finland). HRV indices in the frequency domain were analyzed using the Fast Fourier Transform method and include the following indices: LF (low frequency), HF (high frequency), and the LF/HF ratio (sympathovagal index). The LF/HF ratio was calculated by dividing the LF and HF components, both in ms^2^. Additionally, LF and HF indices underwent correction (removing the very low-frequency component to enhance data specificity) and are expressed in normalized units (n.u.).

For the quantification of the orthostatic test, the 30:15 ratio was calculated promptly after the initiation of the orthostatic period by dividing the RR interval near the 15th heartbeat by the RR interval near the 30th heartbeat. These intervals were evaluated using lead II of the electrocardiogram. The results were considered altered if the 30:15 ratio was <1.04.

Orthostatic hypotension was determined by the variation in blood pressure measurements at the end of the resting period (15 min) and after 3 min of orthostasis (18 min). Altered measurements were defined as a reduction in SBP ≥ 20 mmHg and/or a reduction in diastolic blood pressure (DBP) ≥ 10 mmHg.

### 2.6. Cardiometabolic Risk Markers

Body composition, serum biochemical and inflammatory markers, hemodynamics, and autonomic function are considered cardiometabolic risk markers.

### 2.7. Cardiovascular Risk Scores

Cardiovascular risk was evaluated using specific scores: the atherogenic index of plasma (AIP), atherogenic coefficient (AC), body shape index (ABSI), and Framingham risk score (FR) [10]. The formulas for calculating AIP, AC, and ABSI are shown in Supplementary Table S2.

### 2.8. Statistical Analysis

Results are presented as the mean ± SD or medians [interquartile range (IQR) 25–75] for continuous variables after the Shapiro–Wilk test to assess the distribution of the variables. Categorical variables are presented as numbers (percentages). No formal a priori sample size or power calculation was performed for this secondary analysis. Given the exploratory nature of the study and the relatively small sample size, the analyses were not designed to test predefined primary endpoints but rather to explore associations across multiple cardiometabolic domains. Therefore, the findings should be interpreted as hypothesis-generating. Comparisons of the measures of central tendency were conducted using the Student’s *t*-test (for variables with normal distribution) or the Mann–Whitney test (for variables with non-normal distribution). Associations between categorical variables were assessed using Fisher’s exact test. Associations between NWO and cardiometabolic risk markers and scores were examined using linear regression models for continuous outcomes and logistic regression models for categorical outcomes, with results expressed as beta coefficients (β) or odds ratios (OR), respectively, along with 95% confidence intervals (95% CI). Because of the imbalance in sex distribution between groups, all adjusted models included sex as a covariate; however, residual confounding cannot be ruled out. Given the exploratory nature of this secondary analysis, the relatively small convenience sample, and the number of markers and scores examined, the regression analyses should be interpreted as hypothesis-generating rather than confirmatory. We did not apply formal correction for multiple comparisons; therefore, the findings should be interpreted cautiously, with emphasis on effect estimates, confidence intervals, and the overall consistency of the observed pattern rather than isolated *p*-values alone. The significance level was set at *p* < 0.05. STATA 16 statistical and data science software (Stata Corp, College Station, TX, USA) was used for statistical analyses.

## 3. Results

**Figure 1:**
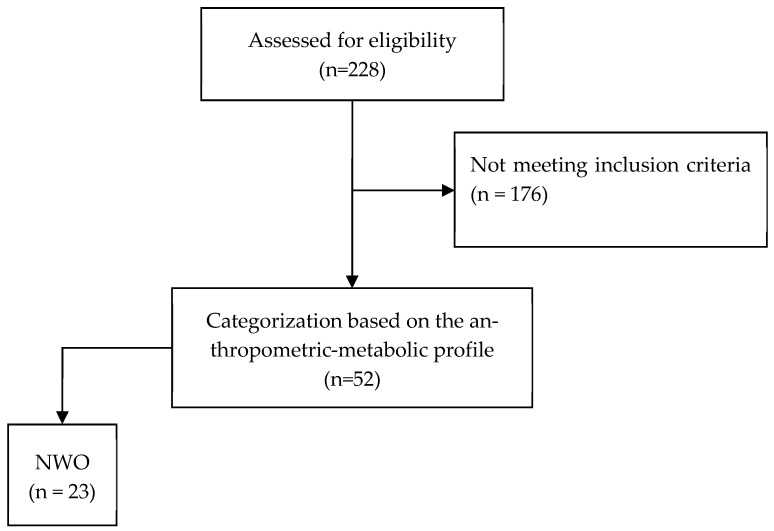
Flowchart of study participants. NWO, normal weight obeseNote: There were no exclusions in the study as the volunteers included in the analysis had all variables filled and complete.

Of the 228 eligible volunteers, 52 met the inclusion criteria and were included in the study. Of these, 29 individuals were classified as having NWNB and 23 as having NWO, as shown in the flowchart in Figure 1.

Table 1 presents the characteristics of the participants included in this study. Overall, the NWO phenotype was predominantly observed in men. NWO showed higher averages of total cholesterol (195.3 ± 30.9 vs. 174.5 ± 33.1 mg/dL; *p* = 0.013), low-density lipoprotein cholesterol (137.0 [117–155] vs. 107.0 [90–127.4] mg/dL; *p* = 0.003), triglycerides (127.0 [90–155] vs. 68.0 [56–85] mg/dL; *p* = 0.001), a higher prevalence of triglyceride alterations (30.4 [7] vs. 0 [0]%; *p* = 0.002), and a lower mean of high-density lipoprotein cholesterol (49.4 ± 17.0 vs. 61.6 ± 17.7 mg/dL; *p* = 0.012). In addition, NWO individuals presented, compared to NWNB, a larger WC (84.6 ± 7.9 vs. 77.3 ± 6.8 cm; *p* = 0.001), HC (100 [97.5–104.5] vs. 96.5 [93–100] cm; *p* = 0.012), and NC (36.1 ± 3.2 vs. 33.8 ± 3.1 cm; *p* = 0.011); higher SBP (124.9 ± 11.8 vs. 116.4 ± 12,9 mmHg; *p* = 0.012), DBP (80.2 ± 6.8 vs. 75.2 ± 9.8 mmHg; *p* = 0.037), and DP (8125 [6804–8906] vs 7140 [6292–7685] mmHg.bpm; *p* = 0.024).

For autonomic function variables, NWO individuals, compared to NWNB, exhibited lower HF (39.1 ± 18.2 vs. 53.2 ± 16.2 n.u.; *p* = 0.005), higher LF (60.7 ± 18.2 vs. 46.6 ± 16.2 n.u.; *p* = 0.005), and a higher LF/HF ratio (1.7 [1–2.9] vs. 1.0 [0.6–1.2]; *p* = 0.005). No significant differences were observed in the 30:15 ratio and OHT when comparing NWO and NWNB (Table 1).

Also, NWO, in comparison to NWNB individuals, presented higher averages of VFA (83.1 [63.5–100.0] vs. 66.1 [51.0–59.9] cm^2^; *p* = 0.006), AC (3.2 [2.4–3.8] vs. 1.9 [1.3–2.7]; *p* <0.001), AIP (0.9 ± 0.5 vs. 0.1 ± 0.5; *p* <0.001), ABSI (7.9 [7.3–8.3] vs. 7.5 [7.2–7.8]; *p* = 0.022), and FR score (5.2 [2.0–10.9] vs. 1.8 [1.0–5.3]; *p* = 0.002) (Table 1).

In the non-adjusted analysis, the NWO phenotype was positively associated with TC (β = +20.8, *p* = 0.025), LDL-c (β = +28.0, *p* = 0.003), and TG (β = +50.4, *p* = 0.001). Moreover, the NWO phenotype was negatively associated with HDL-c levels (β = −12.2, *p* = 0.016). Similarly, the NWO phenotype was associated with body composition (WC: β = +7.2, *p* = 0.001; HC: β = +4.3, *p* = 0.01; and NC: β = +2.2, *p* = 0.015). Observing hemodynamic parameters, in the unadjusted analysis, the NWO phenotype was associated with higher SBP (β = +8.4, *p* = 0.019), DBP (β = +5.0, *p* = 0.041), MAP (β = +6.2, *p* = 0.019), and DP (β = +1020.6, *p* = 0.010). However, after adjustment, these associations were no longer statistically significant (Table 2). Regarding HRV parameters, an association was observed between the NWO phenotype and LF (β: +14.1, *p* = 0.005), HF (β = −14.1, *p* = 0.005), and sympathovagal index (β = +1208.4, *p* = 0.007). Also, NWO was associated with cardiometabolic risk scores such as VFA (β = +24.0, *p* = 0.002) and AIP (β = +0.7, *p* = 0.001) in the unadjusted analysis; however, after adjustment, only VFA and AIP remained associated, whereas ABSI, AC, and Framingham Risk Score were no longer statistically significant (Table 2).

After adjustment for age and sex, NWO remained associated with higher LDL-c, TG, WC, HC, DP, LF, VFA, and AIP, and with lower HF. Associations with SBP, DBP, MAP, ABSI, AC, and Framingham Risk Score were no longer statistically significant after adjustment.

## 4. Discussion

The main finding of the present study was that, among individuals with a normal BMI range, those with a higher body fat percentage showed a less favorable cardiometabolic profile than those with normal body fat percentage. This was evidenced by the association of NWO with an unfavorable lipid profile, altered hemodynamic parameters, autonomic indices, and selected cardiometabolic risk scores, while body composition findings should be interpreted as confirmatory of the phenotype.

For interpretative clarity, the observed findings should be divided into two groups: (1) body composition and anthropometric variables, which are structurally related to the definition of the NWO phenotype and therefore should be interpreted as confirmatory findings, and (2) external clinical markers, such as lipid profile, hemodynamic parameters, and autonomic indices, which provide more independent evidence of cardiometabolic alterations.

The use of BMI to define obesity is limited, as described by Lebiedowska et al. [11], who compared the classification according to BMI with body fat percentage (%BF) measured by bioimpedance. They found that, among nearly 200 women with a BMI considered normal, 26% had a high %BF. Additionally, 3% of women classified as underweight by BMI also had a high %BF [11]. This limitation underscores the need for alternative methods of obesity assessment based primarily on body composition.

Owing to variations in the methods used to assess %BF and its respective cutoff points, both the global and sex-specific prevalence of this condition vary across studies. A meta-analysis from 2022 [12] demonstrated that the prevalence of NWO can vary from 5% to 45% depending on age, sex, and the definition used for NWO. In addition, different ethnic groups present different NWO prevalences, indicating a potential genotypic [13] or sociodemographic [14] component in the prevalence of this condition.

Additionally, it has been demonstrated that the NWO phenotype tends to occur in older individuals [15,16], particularly in women [17]. Jia et al. showed a predominance of 46.5% women in a study with 1147 NWNB individuals in India [7]. This predominance has also been demonstrated in the Japanese [18], Chinese [19], Korean [20], and Colombian [21] populations. However, in our sample, the NWO group was predominantly male, whereas the NWNB group was predominantly female. This marked sex imbalance deserves careful consideration, since sex-related differences in fat distribution, lipid profile, hemodynamics, and autonomic modulation may have influenced part of the observed between-group differences. Although we adjusted the regression models for age and sex, residual confounding cannot be excluded, particularly given the small sample size. Therefore, the independent association between NWO and the studied markers should be interpreted with caution.

### 4.1. Hemodynamics

In the hemodynamic analysis, our study demonstrated that the NWO group had higher mean SBP, DBP, MAP, and double product than the NWNB group, which aligns with current publications on this subject. A study conducted in a Chinese population reported that individuals with NWO were more likely to present hypertension compared to individuals who are overweight but have a low %BF [22]. A similar result was found by Correa-Rodríguez et al., who demonstrated an increased risk of hypertension in the NWO group [21].

### 4.2. Lipid Profile and Anthropometric Evaluation

The lipid profile of NWO demonstrates significantly higher mean serum levels of TC and TG and significantly lower mean serum levels of HDL-c than those of NWNB, resembling a pattern of an unfavorable cardiometabolic profile in this population [23]. Kapoor et al. [7] found a significantly elevated odds ratio for dyslipidemia in the NWO group compared to the NWNB group (OR = 2.37). Additionally, Kim et al. [24] conducted a study with 291 men and 1281 women with the NWO phenotype and found significant alterations in LDL, HDL, and TG in both groups compared to those in the NWNB group.

Regarding anthropometric evaluation on NWO, Madeira et al. [25] describe in their study changes in WC (WC) (80.5 vs. 87.7 in men and 71.6 vs. 77.3 in women), as well as HC (HC) (96.9 vs. 101.2) when comparing NWO with NWNB [25]. These anthropometric findings should also be interpreted cautiously, as they are closely related to the body composition phenotype itself. The increase in these anthropometric variables has also been reported in other studies [20,21,24]. Currently, there is no literature associating NWO with larger NCs; however, individuals with overweight and obesity exhibit larger NCs [26]. A study demonstrated a higher incidence of cardiovascular events in participants with a high NC compared to individuals with a low NC, especially in men [27].

### 4.3. Autonomic Function

Chronic hyperactivity of the sympathetic nervous system contributes to reduced insulin sensitivity and is associated with the development of hypertension and metabolic syndrome, increasing the risk of cardiovascular and renal diseases [28]. Reduced values of heart rate variability (HRV) have been shown to be a strong predictor of low survival in patients with left ventricular dysfunction, heart failure, or previous acute myocardial infarction [29], and are associated with hyperglycemia, hypertension, dyslipidemia, presence of type 2 diabetes, high body mass index (BMI), and cardiovascular diseases [30].

Izumi et al. [31] studied postpartum women with normal weight obesity and their weight loss after childbirth. Although there was an increase in the means of low-frequency (LF), high-frequency (HF), and total power during the one-year weight loss, the results were not statistically significant. In line with this, Araujo et al. [32] found no correlation between body fat percentage (%BF) and HRV in individuals with normal weight, overweight, and obesity. By contrast, Triggiani et al. [33] found a reduction in the frequency domain values, indicating impaired sympathetic cardiac activity in overweight women compared to normal-weight women. Espinoza-Salinas et al. [5] demonstrated similar results to ours, comparing individuals with normal weight, overweight, and obesity, revealing an increase in the LF component and a reduction in the HF component in overweight individuals and especially in the obese.

Although the consistently observed association of NWO with poor HRV indices, HRV analysis has inherent limitations due to participants’ behavioral and individual heterogeneity. To minimize these biases during ESAT study, RR interval recordings for HRV analysis were made after blood collection following a 12-h fast. Also, volunteers were instructed to avoid caffeine and alcohol consumption, ensure 8 h of sleep, and refrain from intense physical activity in the prior 24 h prior to the exam. Nonetheless, full adherence remains challenging, particularly given the effects of smoking. Importantly, HRV measurements are highly sensitive to uncontrolled factors such as caffeine and nicotine intake, recent physical activity, sleep quality, breathing pattern, and medication use. These factors may have influenced LF, HF, and LF/HF results, thereby limiting confidence in the autonomic findings.

### 4.4. Cardiometabolic Risk Scores

Our results demonstrate that the NWO phenotype is associated with higher visceral adiposity than the NWNB phenotype, which is consistent with the defining characteristics of the phenotype and should therefore be interpreted as a confirmatory rather than an independent finding. Premenopausal women tend to store fat subcutaneously, whereas men and postmenopausal women tend to accumulate visceral fat [34]. In one study, it was demonstrated that NWO individuals had a higher percentage of body fat and higher values of subcutaneous and visceral fat, which supports the structural relationship between the phenotype definition and these body composition findings. Furthermore, the amount of visceral fat is an independent risk factor for subclinical coronary atherogenesis [16]. Similarly, in a larger study conducted in Colombia, it was shown that young NWO adults presented higher values of visceral fat, which were associated with an increased risk of abdominal obesity [21].

In our analysis, it was evident that the NWO group exhibited a higher median than the NWNB group. Ma et al. [35] demonstrated that ABSI is a predictor of subclinical carotid atherosclerosis, even in patients without prior comorbidities. This index is positively associated with all-cause mortality; individuals with high ABSI have approximately a 30% higher risk of death than those with low ABSI within each BMI category [36]. Additionally, in the original ABSI article, a higher ABSI value is associated with greater abdominal fat deposition [37].

Our results demonstrate that NWO individuals have a higher mean AIP than NWNB individuals. Additionally, NWO was associated with higher AIP values than NWNB in our regression analysis. To date, no studies have linked AIP to increased cardiovascular risk in NWO individuals.

A study correlated cardiovascular risk predictors in overweight or centrally obese individuals with familial hypercholesterolemia [38] and demonstrated that both groups exhibited increased AIP compared to the group without elevated BMI or WC. Shen et al. [10], demonstrated that elevated AIP values were found in individuals with abdominal obesity compared to those without this condition, associating increased WC with increased AIP.

In addition, Chhezom et al. [39] found that AIP showed a progressively higher value in individuals with normal BMI, overweight, and obesity (0.053 (0.091–0.120); 0.281 (0.038–0.476); 0.331 (0.167–0.448), respectively). Furthermore, a study conducted in Taiwan, with obesity defined as a BMI ≥ 27 kg/m^2^, showed that individuals with high AIP levels tend to have a higher risk of obesity [40]. The NWO group exhibited a higher median AC than the NWNB group.

Previous studies have demonstrated that the non-HDL-C/HDL-C ratio is associated with atherosclerotic plaque formation [41]. In a cohort conducted in Greece, it was shown that individuals without comorbidities who experienced cardiovascular events over a 10-year period had higher non-HDL/HDL cholesterol ratios than those who did not experience the event [42]. Similarly, the literature has demonstrated that individuals with coronary atherosclerosis who underwent coronary artery bypass graft surgery had significantly higher AIP and AC than individuals without coronary atherosclerosis [43].

Park Y [44] found that in a study, 640 individuals with normal BMI but high WC, where this group had a FR of 7%, while the group with normal BMI and WC had only 5.4%. It has also been shown that individuals with normal BMI but with sarcopenia also had increased FR compared to individuals with normal BMI but without sarcopenia [45].

Our results demonstrated that individuals with NWO had a higher median Framingham Risk Score than those with NWNB; however, both groups remained within the low-risk range (<10%). Therefore, this finding should be interpreted as a difference in estimated risk score distribution within a low-risk sample, rather than evidence of high absolute cardiovascular risk or future events. Our findings should be interpreted as indicating cross-sectional differences in risk markers rather than evidence of future progression to moderate or high cardiovascular risk. Taken together, these findings support the view that normal-weight obesity is associated with an unfavorable cardiometabolic profile not captured by BMI alone. However, our data do not allow conclusions regarding future clinical outcomes or broader functional consequences [46].

This study has important limitations that should be considered when interpreting the findings. First, this was a cross-sectional secondary analysis based on a relatively small convenience sample. Second, participants were recruited from a single public cardiology hospital, and the sample consisted exclusively of hospital workers, which may limit external validity. This occupational group may differ from the general population in terms of health awareness, work-related stress, access to care, and lifestyle patterns. Therefore, the present findings should not be generalized without caution to broader community-based populations, other occupational groups, or populations with different sociodemographic characteristics.

Additionally, the relatively small sample size in relation to the number of evaluated outcomes increases the risk of unstable estimates and chance findings. Importantly, the study was not powered to detect differences across all evaluated outcomes, and no predefined primary endpoints were established. This further reinforces the exploratory nature of the analyses.

Moreover, some body composition and anthropometric outcomes are structurally related to the definition of the NWO phenotype and should therefore be interpreted as confirmatory rather than independent findings. In addition, autonomic results should be interpreted cautiously because HRV measurements may have been influenced by uncontrolled behavioral and physiological factors.

Despite these limitations, our findings contribute to the growing body of evidence suggesting that body mass index alone may be insufficient to capture relevant cardiometabolic alterations. In line with this perspective, recent high-impact discussions in the obesity field have reinforced the importance of moving beyond BMI-only classifications when interpreting metabolic risk, while also highlighting the need for more precise phenotyping and outcome-based validation of obesity-related risk categories [47]. These findings should be interpreted as hypothesis-generating and require confirmation in larger, prospective studies with more comprehensive adjustment for confounders.

## 5. Conclusions

In this sample of hospital workers with normal BMI, NWO was associated with worse cardiometabolic markers and selected cardiometabolic risk scores than NWNB. These findings suggest that excess body fat, even in the presence of normal BMI, is associated with an unfavorable cardiometabolic profile. However, because of the cross-sectional design, secondary nature of the analysis, and limited sample size, no causal or prognostic inference can be made. Prospective studies are needed to determine whether NWO predicts future cardiometabolic events.

## Figures and Tables

**Table 1:** Characteristics of participants included in the study, stratified by NWO and NWNB.

Variables	NWNB (N = 29) Mean ± SD or Median [IQR 25–75] or Percentage (N)	NWO (N = 23) Mean ± SD or Median [IQR 25–75] or Percentage (N)	*p*-Value
Age (years)	42.6 ± 11.9	44.2 ± 14.8	0.890
Sex			
Male	8 (27.6)	16 (69.6)	0.003
Female	21 (72.4)	7 (30.4)	
Cardiometabolic Risk Markers			
Lipid Profile			
TC (mg/dL)	174.5 ± 33.1	195.3 ± 30.9	0.013
LDL-c (mg/dL)	107.0 [90.0–127.4]	137.0 [117.0–155.0]	0.003
HDL-c (mg/dL)	61.6 ± 17.7	49.4 ± 17.0	0.012
TG (mg/dL)	68.0 [56.0–85.0]	127.0 [90.0–155.0]	0.001
TG > 150 mg/dL, n (%)	0 (0)	30.4 (7)	0.002
Fasting Glucose (mg/dL)	87.0 [80.0–92.0]	88 [85.0–94.0]	0.280
CRP (mg/dL)	0.1 [0.0–0.1]	0.1 [0–0.3]	0.399
Circumferences			
WC (cm)	77.3 ± 6.8	84.6 ± 7.9	0.001
HC (cm)	96.5 [93.0–100.0]	100.0 [97.5–104.5]	0.012
NC (cm)	33.8 ± 3.1	36.1 ± 3.2	0.011
Hemodynamics			
SBP (mmHg)	116.4 ± 12.9	124.9 ± 11.8	0.012
DBP (mmHg)	75.2 ± 9.8	80.2 ± 6.8	0.037
MAP (mmHg)	89.0 ± 10.4	95.1± 7.2	0.009
DP (mmHg.bpm)	7140 [6292–7685]	8125 [6804–8906]	0.024
Autonomic Function			
LF (n.u.)	46.6 ± 16.2	60.7 ± 18.2	0.005
HF (n.u.)	53.2 ± 16.2	39.1 ± 18.2	0.005
Sympathovagal Index	1.0 [0.6–1.2]	1.7 [1.0–2.9]	0.005
Altered 30:15 ratio	10 (34.5)	4 (18.2)	0.165
Orthostatic Hypotension	4 (13.8)	5 (21.7)	0.349
Cardiometabolic Risk Scores			
VFA (cm^2^)	66.1 [51.0–69.9]	83.1 [63.5–100.0]	0.006
ABSI	7.5 [7.2–7.8]	7.9 [7.3–8.3]	0.022
AIP	0.1 ± 0.5	0.9 ± 0.5	<0.001
AC	1.9 [1.3–2.7]	3.2 [2.4–3.8]	<0.001
Framingham Risk Score (%)	1.8 [1.0–5.3]	5.2 [2.0–10.9]	0.002

TC, total cholesterol; LDL-c, low-density lipoprotein cholesterol; HDL-c, high-density lipoprotein cholesterol; TG, triglycerides; CRP, C-reactive protein; WC, waist circumference; HC, hip circumference; NC, neck circumference; SBP, systolic blood pressure; DBP, diastolic blood pressure; MAP, mean arterial pressure; DP, double product; LF, low frequency; HF, high frequency; VFA, visceral fat area; ABSI, A Body Shape Index; AIP, atherogenic index of plasma; AC, atherogenic coefficient.

**Table 2:** Association of NWO phenotype with cardiovascular risk markers and scores.

Variables	β or OR	95% CI	*p*-Value	β or OR	95% CI	*p*-Value
	Non-Adjusted			Adjusted for Age and Sex		
Lipid Profile						
TC (mg/dL)	+20.8	+2.7–+38.8	0.025	+19.4	+0.0–+38.9	0.050
LDL-c (mg/dL)	+28.0	+9.8–+46.2	0.003	+23.6	+3.7–+43.6	0.021
HDL-c (mg/dL)	−12.2	−21.9–−2.4	0.016	−6.4	−16.8–+3.9	0.216
TG (mg/dL)	+50.4	+30.4–+70.5	<0.001	+45.7	+23.6–+67.7	<0.001
Fasting Glucose (mg/dL)	+6.0	−3.4–+15.5	0.206	+5.2	−5.3–+15.7	0.322
CRP (mg/dL)	+0.1	−0.2–+0.3	0.506	+0.1	−0.2–+0.4	0.602
Body Composition						
WC (cm)	+7.2	+3.1–+11.3	0.001	+4.0	+0.1–+8.0	0.046
HC (cm)	+4.3	+1.0–+7.54	0.011	+5.0	+1.4–+8.6	0.008
NC (cm)	+2.2	+0.4–+4.0	0.015	−0.2	−1.4–+1.0	0.736
Hemodynamics						
SBP (mmHg)	+8.4	+1.4–+15.4	0.019	+3.7	−3.3–+10.7	0.297
DBP (mmHg)	+5.0	+0.2–+9.9	0.041	+3.3	−1.9–+8.6	0.211
MAP (mmHg)	+6.2	+1.0–+11.3	0.019	+3.4	−1.9–+8.8	0.205
DP (mmHg.bpm)	+1020.6	+250.5–+1790.7	0.010	+1083.1	+218.5–+1947.7	0.015
Autonomic Function						
LF (nu)	+14.1	+4.4–+23.9	0.005	+11.4	+1.7–+21.1	0.023
HF (nu)	−14.1	−23.8–−4.3	0.005	−11.4	−21.1–−1.7	0.022
Sympathovagal Index	+1208.4	+352.1–+2064.6	0.007	+910.2	−8.6–+1829.1	0.052
Altered 30:15 ratio	+0.4	+0.1–+1.6	0.203	+0.5	−0.1–+2.3	0.406
Orthostatic Hypotension	+1.7	+0.4–+7.4	0.455	+1.7	+0.3–+8.2	0.528
Cardiometabolic Risk Scores						
VFA (cm^2^)	+24.0	+9.4–+38.5	0.002	+30.2	+14.7–+45.707	<0.001
ABSI	+0.4	+0.1–0.6	0.019	+0.2	−0.1–+0.5	0.132
AIP	+0.7	+0.4–+1.0	<0.001	+0.6	+0.2–0.9	0.001
AC	+1.6	+0.3–+3.0	0.018	+1.1	−0.3–+2.6	0.128
Framingham Risk Score (%)	+4.3	−0.4–+9.0	0.070	+0.7	−2.6–+4.1	0.657

Values are presented as β coefficients for continuous outcomes and odds ratios (OR) for categorical outcomes, with respective 95% confidence intervals (95% CI). Models are shown as crude and adjusted for age and sex. Abbreviations: TC, total cholesterol; CRP, C-reactive protein; WC, waist circumference; HC, hip circumference; NC, neck circumference; SBP, systolic blood pressure; DBP, diastolic blood pressure; MAP, mean arterial pressure; DP, double product; VFA, visceral fat area; ABSI, A Body Shape Index; AIP, atherogenic index of plasma; AC, atherogenic coefficient.

## Data Availability

The data presented in this study are available on request from the corresponding author due to ethical and privacy restrictions.

## Supplementary Materials

The following supporting information can be downloaded at: https://www.mdpi.com/article/10.3390/healthcare14081008/s1, Table S1. Expected Body Fat Percentage According to Sex and Age Range. Table S2. Cardiovascular Risk Scores.

## Abbreviations

The following abbreviations are used in this manuscript:

AC	Atherogenic Coefficient
AIP	Atherogenic Index of Plasma
ANS	Autonomic Nervous System
ABSI	A Body Shape Index
BMI	Body Mass Index
CRP	C-Reactive Protein
DBP	Diastolic Blood Pressure
DP	Double Product
ESAT	Study of Workers’ Health (Estudo de Saúde do Trabalhador)
FR	Framingham Risk Score
HC	Hip Circumference
HDL-c	High-Density Lipoprotein Cholesterol
HF	High Frequency (Heart Rate Variability)
HRV	Heart Rate Variability
IL-6	Interleukin-6
LDL-c	Low-Density Lipoprotein Cholesterol
LF	Low Frequency (Heart Rate Variability)
MAP	Mean Arterial Pressure
NC	Neck Circumference
NIC	National Institute of Cardiology
NWNB	Normal Weight with Normal Body Fat
NWO	Normal Weight Obesity
OHT	Orthostatic Hypotension Test
RR	R–R Interval
SBP	Systolic Blood Pressure
TC	Total Cholesterol
TG	Triglycerides
TNF-α	Tumor Necrosis Factor Alpha
VFA	Visceral Fat Area
WC	Waist Circumference
WHO	World Health Organization
