# Supplementary material for: Normal-Weight Obesity and an Unfavorable Cardiometabolic Profile: Results from the Study of Workers’ Health (ESAT)
Source: Healthcare (Basel). 2026 Apr 11;14(8):1008. doi: 10.3390/healthcare14081008 (PMC13116948; doi:10.3390/healthcare14081008)
Supplement: Supplementary file 1 [file healthcare-14-01008-s001.zip › healthcare-4239275-supplementary.pdf]

Table S1. Expected Body Fat Percentage According to Sex and Age Range.

Sex	20 – 39 years	40 – 59 years	60 – 79 years
Male	≤ 19.9 %BF	≤ 21.9 %BF	≤ 24.9 %BF
Female	≤ 32.9 %BF	≤ 33.9 %BF	≤ 35.9 %BF

Adapted from Kosmala et al. (2012). %BF = Body Fat Percentage

Table S2. Cardiovascular Risk Scores.

AIP = $log_{10}^{\frac{TG}{HDL-c}}$
AC = $\frac{non-HDL-c}{HDL-c}$
ABSI = $\frac{Waist\ Circunference}{\sqrt[3]{BMI^2} * \sqrt[2]{Height}}$

AIP, atherogenic index of plasma; AC, atherogenic coefficient; ABSI, body shape index; TG, triglycerides; HDL-c, high-density lipoprotein; BMI, body mass index.
